# Acute intestinal GVHD following donor-derived CD7-CAR-T-cell infusion in a child with Omicron COVID-19

**DOI:** 10.1097/BS9.0000000000000170

**Published:** 2023-11-02

**Authors:** Yu Lian, Zhilin Gao, Juanjuan Ti, Zhuanzhuan Yu, Liangming Ma, Jia Wei

**Affiliations:** a Department of Hematology, Shanxi Bethune Hospital, Shanxi Academy of Medical Sciences, Tongji Shanxi Hospital, Third Hospital of Shanxi Medical University, Taiyuan 030032, China; b Tongji Hospital, Tongji Medical College, Huazhong University of Science and Technology, Wuhan 430030, China

## 1. INTRODUCTION

CD7 is an ideal chimeric antigen receptor (CAR) target for T-cell acute lymphocytic leukemia (T-ALL). Donor-derived CAR-T-cell therapy, as an emerging treatment strategy, shows excellent efficacy in refractory/relapsed (r/r) T-ALL, with over 90% of complete remission (CR), brings new promise to improve prognosis and survival quality, and provides more opportunities for following bridging transplantation.^1^ However, CD7-CAR-T-cell recipients are always immunocompromised for a number of reasons: depletion of healthy T and NK cells; the need for drugs, which are lymphodepleting, prior to CAR-T-cell therapy; a history of hematological malignancies; multiple-lines chemotherapy; and hematopoietic stem-cell transplantation (HSCT). Meanwhile, patients post donor-derived CAR-T-cell therapy are at high risk of graft-versus-host disease (GVHD) considering infusion of allogeneic cell and increase incidence of infection.^2–5^

Since the beginning of coronavirus disease 2019 (COVID-19) pandemic in 2020, the increasing transmissibility of the SARS-Cov2 virus and probable cytokines release syndromes (CRS), undoubtedly pose a huge challenge to safety of immunotherapy, especially in patients who received donor-derived CAR-T-cell therapy.^6^ For those who have been infected with the SARS-Cov-2, CAR-T-cell treatment should be put off until 2 weeks after the relief of COVID-19 and reverse of negative in RNA test.^7,8^ However, for patients already undergoing CAR-T-cell treatment, it is not yet known whether the development of a COVID-19 infection will increase the risk of CRS or GVHD or their severity, or whether the immunodeficiency caused by CD7-CAR-T-cell therapy would worsen the COVID symptoms.

We report here, at first time that a T-ALL child who relapsed after allogenic-HSCT was treated with donor-derived CD7-CAR-T-cell therapy, accompanied by Omicron variant infection and subsequently soon severe intestinal acute GVHD (aGVHD). The case provided aims to serve as a reference for the clinical application and safety evaluation of donor-derived CD7-CAR-T-cell therapy in r/r T-ALL associated with COVID-19 diseases.

## 2. CASE REPORTS

**Figure 1.**
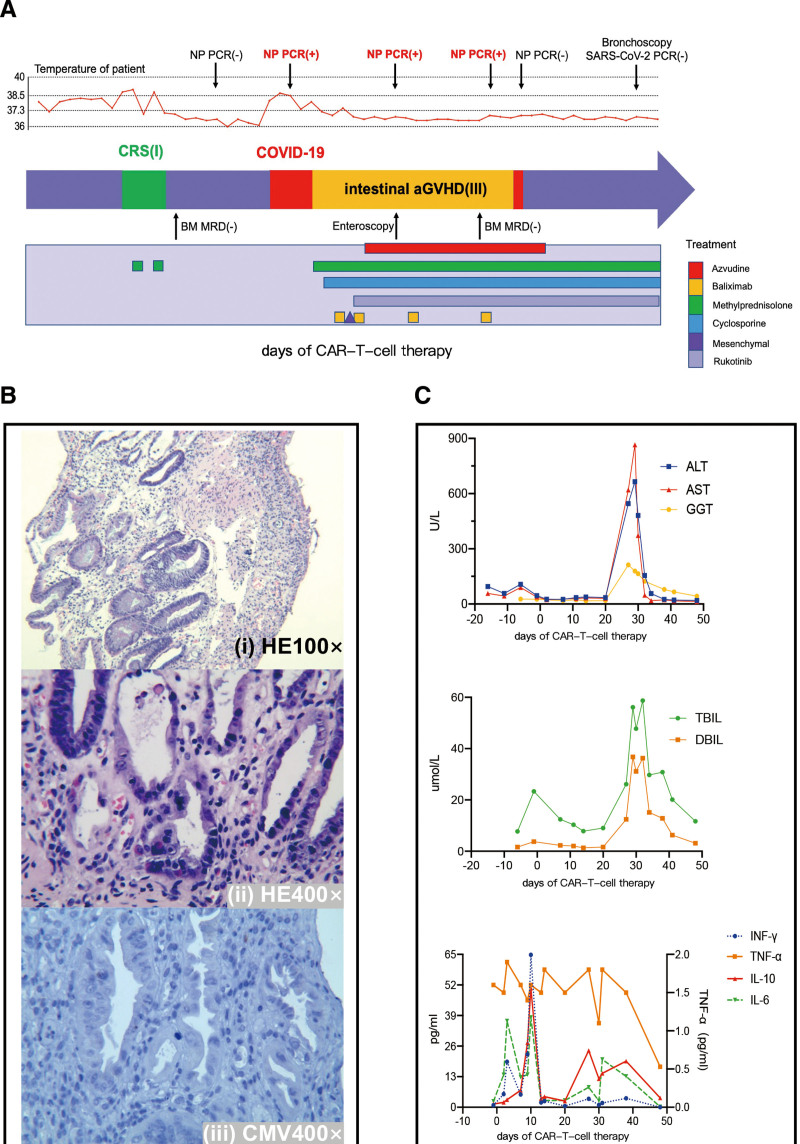
Summary of clinical courses, treatment and laboratory results for the patient after donor-derived CD7-CAR-T-cell therapy. (A) Clinical timeline of disease course and key clinical information post the CAR-T-cell infusion. (B) Tissue sections of the enteroscopic biopsy demonstrated that predominant chronic inflammation of mucosa and no infection of cytomegalovirus. (C) Changes in liver enzymes and cytokines reflected the hallmarks of aGVHD and CRS after CAR-T-cell therapy. aGVHD = acute graft-versus-host disease, ALT = alanine aminotransferase, AST = aspartate aminotransferase, CAR = chimeric antigen receptor, CMV = cytomegalovirus, COVID-19 = coronavirus disease 2019, CRS = cytokine release syndrome, DBIL = direct bilirubin, GGT = gamma-glutamyl transpeptidase, IFN = interferon, IL = interleukin, NP = nasopharyngeal swab, PCR = polymerase chain reaction, SARS-Cov2 = severe acute respiratory syndrome coronavirus 2, TBIL = total bilirubin, TNF = tumor necrosis factor.

An 11-year-old girl was diagnosed with T-ALL with gene mutations including PTEN, EED, FAT1 and CUX1 in July 2020 and had undergone induction chemotherapy to achieved remission followed with multiple-lines consolidation therapy. On November 16, 2021, the patient received haploidentical HSCT (father, 6/12 HLA match). In October 2022, morphological evaluation of bone marrow showed 68.5% lymphoblasts and prolymphocytes, and short tandem repeat (STR) polymerase chain reaction analysis demonstrated 36.4% of donor chimerism, indicating the relapse of ALL. With a conditioning regimen of fludarabine (30 mg/m^2^, 3 days) and cyclophosphamide (300 mg/m^2^, 3 days), donor-derived CD7-CAR-T-cell (5 × 10^5^ cells/kg) was infused on November 11, 2022 (d0). Nine days later, grade 1 CRS occurred but was immediately controlled with methylprednisolone (1 mg/kg, day 10 and 12 post CAR-T-cell infusion) (Fig. 1A, Supplemental table 1, http://links.lww.com/BS/A74).

With the pandemic wave of Omicron variant in China in December 2022,^9^ the child was unvaccinated before and identified as being positive for SARS-COV2 virus at day 25 after CAR-T-cell therapy. Cycle thresholds were 28.05 and 27.29 for the OFR1ab and N genes, respectively. First, the patient felt febrile and muscular ache, with normal oxygen saturation on room air and clear lungs by computed tomography. Unfortunately, 3 days after infection, the child had intermittent abdominal pain, diarrhea, and rapidly developed into persistent bloody stool. Laboratory evaluation showed a significant increase in transaminase and bilirubin. The child was confirmed acute GVHD (aGVHD: gut, grade III; liver, grade III). Pathological findings at enteroscopic biopsy revealed chronic inflammation of mucosa and a decreasing number of crypts including some with atypical shapes, which were consistent with aGVHD. The specimen also showed that epithelial cells were of enlarged nuclei with evident nucleoli, indicating viral infection. However, immunochemical staining demonstrated that no cytomegalovirus, Epstein-Barr virus and polyomavirus were found (Fig. 1A–B). The specimens were further for metagenomic next-generation sequencing (mNGS), which supported a diagnosis of *Enterococcus faecium*, but no Omicron variant.

**Figure 2.**
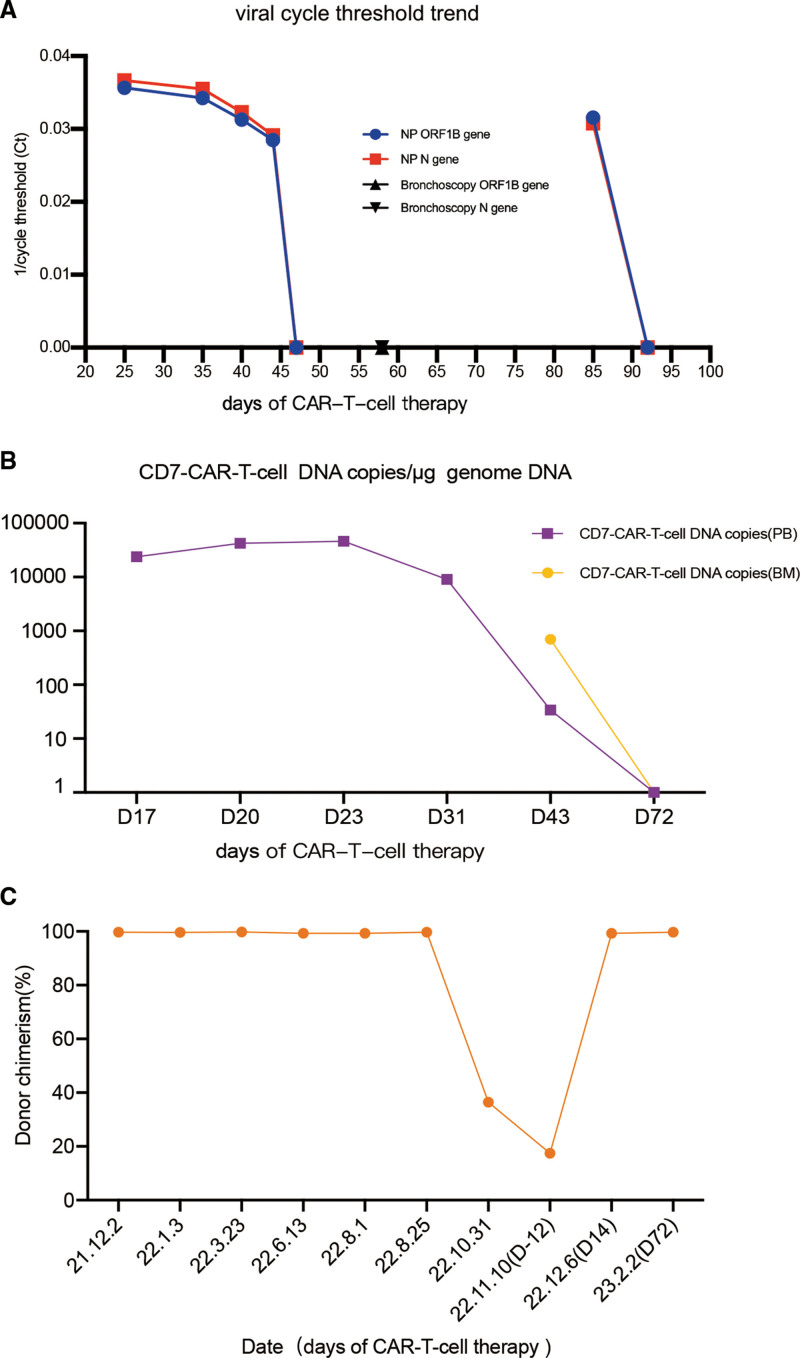
The safety and efficacy of donor-derived CD7-CAR-T-cell therapy with SARS-CoV-2 infection. (A) Patients’ SARS-CoV-2 viral cycle threshold trend indicated the severely delayed virus clearance and again positive virus test later. (B) Dynamics analysis of CD7-CAR-T-cell revealed excellent amplification and DNA copies of CAR-T-cell transgenes increased up to about 5 × 10^4^. (C) Donor chimerism rate of the patient showed that STR-PCR resolved to fully donor chimerism again after CAR-T-cell therapy. BM = bone marrow, N = nucleocapsid protein, OF = open-reading frame, PB = peripheral blood, STR-PCR = short tandem repeat polymerase chain reaction.

With administration of methylprednisolone and cyclosporine (maintenance concentration, 150–250 μg/L), transaminase and bilirubin resolved rapidly but intestinal aGVHD failed to be controlled. The child was then treated with basiliximab (20 mg, twice a week for 2 weeks), mesenchymal stromal cells (once in 31 days after infusion, not continued due to the economic burden) and JAK inhibitor ruxolitinib (10mg twice daily). The methylprednisolone was administrated by 2 mg/kg/d for a week and reduced by 10 to 20 mg every 5 to 7 days as other medicines above added and the condition improved. In the meantime, Azvudine (FNC) was administered for reducing viral load of SARS-CoV-2. However, the virus test of nasopharyngeal swabs could not turn negative until 22 days later. We further conducted the RNA test on a lower respiratory tract lavage fluid later and luckily found no positive result (Fig. 2A). The GVHD was controlled with mushy stool of 150 to 300 mL once a day but no more bloody stool or abdominal pain.

Encouragingly, the dynamic analysis of CD7-CAR-T-cell therapy showed excellent amplification in both peripheral blood and bone marrow. Copies of CAR-T-cell transgenes increased up to about 5 × 10^4^. Consequently, the child reversed to CR again. On 14, 43, and 72 days after CAR-T-cell therapy, bone marrow and flow cytometry indicated CR (Supplemental Figure 1, http://links.lww.com/BS/A74), and STR resolved to fully donor chimerism (Fig. 2B–C). So, the patient could be discharged home transiently for regular follow-up and following treatment. The neutrophil lasts to be greater than 0.5 × 10^9^/L and the patient lives without blood transfusion.

Nevertheless, 85 days post CAR-T-cell treatment at home, the child’s omicron nucleic acid showed positive again, accompanied by mild fever and diarrhea. The patient immediately began taking Paxlovid medication. Fortunately for this time the nucleic acid turned negative after 7 days due to regular monitoring and timely treatment.

## 3. DISCUSSION

Despite lower lethal risks in former healthy persons, Omicron with its faster spread and stronger ability to escape vaccine-induced immunity, still brought enormous threats for patients with immunodeficiency. The recent study about hematologic patients infected with omicron showed 8.2% of critical illness which were admitted to an intensive care unit occurred. For adult hematological tumor with CAR-T-cell therapy or HSCT, mortality from SARS-CoV-2 infections has been reported as 33% to 40%; An international study of CAR-T-cell recipients between 0 and 30 years with SARS-CoV-2 infections showed a 28.6% hospitalization rate and 4.3% death rate.^9–13^ However, the impact of Omicron infection on patients with CD7-donor-derived CAR-T-cell treatment is not well understood.

Donor-derived CD7-CAR-T-cell therapy has great potential in r/r T-ALL.CD7 is expressed on the surface of normal and over 95% of T-ALL T cells, respectively. Furthermore, donor-derived CD7-CAR-T-cell solves the problems of fratricide and malignant contamination by autologous CD7-CAR-T-cell.^14,15^ In a clinical study of 20 patients with r/r T-ALL, donor-derived CD7-CAR-T-cell has achieved 90% of CR with manageable safety profile. Another research of 20 patients with r/r T-ALL and lymphoblastic lymphoma showed the similar results after CD7-CAR-T-cell therapy, with about 94.12% CR and no serious adverse events.^2,3^

It could not inevitably ignore the high risk of aGVHD in CD7-CAR-T-cell therapy since the donor nature of CAR-T-cell. Li et al reported a study of 12 patients of r/r T-ALL or T-cell lymphoblastic lymphoma who underwent donor CD7-CAR-T-cell therapy then bridging to allo-HSCT, most of whom had active GVHD after CAR-T-cell therapy and 3 developed aGVHD following allo-HSCT. In some other studies about donor-derived CD7-CAR-T-cell, the incidence of GVHD was over 50%.^2–4^

Omicron may further exacerbate the risk of gut GVHD. GVHD is the immune-mediated tissue damage by the activation of donor immune-competent T cells with antigens normally expressed by the recipient. Host exposure to gut microbiota due to gut barrier disruption is one of the important initiating events for GVHD.^16^ Several studies have found that a considerable number of COVID-19 patients presented with gastrointestinal symptoms; and SARS-CoV-2 virus was found in the fecal specimens of more than half patients. Research studies show that the gastrointestinal changes might be the secondary effect of respiratory changes, finally resulting in the imbalance of intestinal ecological. Intestinal cells highly express angiotensin-converting enzyme 2 and transmembrane protease serine 2 receptors, which are targets of SARS-CoV-2 and play a vital role in entry for viral infection of gastrointestinal cells^.17–20^ On the other hand, previous studies have shown that the SARS-CoV-2 vaccine may be a potential trigger of GVHD after transplantation. In a study of 113 allo-HSCT patients, 13% developed new or exacerbated GVHD symptoms after vaccination. In another study of 298 patients with allo-HSCT, the overall incidence of new chronic GvHD and exacerbated chronic GvHD after receiving the SARS-CoV2 vaccine was 14%, and the median time from vaccination to GVHD was about 3 to 4 weeks. In multivariate analysis, previous chronic GVHD and recent transplantation are associated with a higher incidence of GVHD after COVID-19 vaccination^.21,22^

In this case, the child experienced COVID-19 pandemic during CD7-donor-derived CAR-T-cell treatment. The child contracted Omicron and developed severe intestinal GVHD in 3 days. Because it was less than 1 month after CAR-T-cell treatment at that time, the child was well cared from both the hospital and the parents without unclean diet, dietary change, or other special cases. What is more, the intestinal biopsy confirmed the existence of GVHD, and more importantly the highly suspicious virus infection by altered epithelium with its thickened capsule, enlarged nuclei, and prominent nucleoli. Further immunohistochemistry revealed that this viral infection was not cytomegalovirus, Epstein-Barr virus, or polyomavirus. We also sent intestinal specimens for mNGS but did not find definitive evidence of SARS-CoV-2 maybe due to some omissions in unstable RNA virus for sample storage and transportation problems. Since the virus is ubiquitous and will continue for a long time, considering its significant impact on the severe GVHD and prognosis of patients after CAR-T-cell especially CD7-CAR-T-cell therapy, the subtle relationship between COVID-19 and GVHD should be given sufficient attention and further research.

For patients with severe immunodeficiency after CAR-T-cell therapy, it was not easy to eliminate COVID-19, and combination of severe GVHD undoubtedly further aggravated the difficulty. We quickly implemented intensive antiviral treatment and anti-GVHD treatment. Considering the interaction between plaxivoid and other drugs including cyclosporine, we chose the available treatment Azivudine for anti-virus.^23^ The COVID-19 might result in more severe intestinal rejection, whereas the treatment of anti-GVHD may also avoid the serious CRS reaction of the lungs and even the whole body that COVID-19 may induce. Luckily, the covid-19 diseases was relieved and the intestinal GVHD was also well controlled. So, regular SARS-CoV-2 RNA testing and timely and effective antiviral treatment are urgent, which may help to change the extremely dangerous phenomenon into a manageable and benefit outcome for patients.

In conclusion, this is the first time to depict the course of donor-derived CD7-CAR-T-cell patients after infection with omicron, which has considerable clinical implications for how to treat these patients more safely in the future. Patients after CD7-CAR-T-cell treatment who are infected with Omicron may experience severe delayed virus clearance and multiple relapses, exacerbating the risk of GVHD and pneumonia. Therefore, during the prevalence of COVID-19 diseases in local areas, donor-derived CD7-CAR-T-cell should be performed cautiously or even postponed if conditions permit. Although omicron no longer poses a threat in the normal population, more data and further exploration are needed on how to manage patients receiving CD7-CAR-T-cell treatment during local COVID-19 outbreaks.

## ACKNOWLEDGMENT

This study was supported by a grant from the National Natural Science Foundation of China (No 82070217).

The authors acknowledge the team of staff and researchers at the department of hematology and clinical laboratory center of Shanxi Bethune Hospital for their assistance.

## AUTHOR CONTRIBUTIONS

J.W. and Z.G. conceived of the study. Y.L. and J.T. collected and analyzed the clinical data. L.M. and Z.Y. contributed to data collection, diagnosis, and treatment of the diseases. Y.L., Z.G., and J.W. wrote the manuscript with the help of all the authors.

## Supplementary Material



## References

[1] Wei W Yang D Chen X Liang D Zou L Zhao X. Chimeric antigen receptor T-cell therapy for T-ALL and AML. Front Oncol. 2022;12:967754. 36523990 10.3389/fonc.2022.967754 PMC9745195

[2] Pan J Tan Y Wang G. Donor-derived CD7 chimeric antigen receptor T cells for T-cell acute lymphoblastic leukemia: first-in-human, phase I trial. J Clin Oncol. 2021;39(30):3340–3351. 34324392 10.1200/JCO.21.00389

[3] Lu P Liu Y Yang J. Naturally selected CD7 CAR-T therapy without genetic manipulations for T-ALL/LBL: first-in-human phase 1 clinical trial. Blood. 2022;140(4):321–334. 35500125 10.1182/blood.2021014498

[4] Li Z An N Yang K. Donor CD7 chimeric antigen receptor T cell bridging to allogeneic hematopoietic stem cell transplantation for T cell hematologic malignancy. Transplant Cell Ther. 2023;29(3):167–173. 36427783 10.1016/j.jtct.2022.11.013

[5] Vora SB Waghmare A Englund JA Qu P Gardner RA Hill JA. Infectious complications following CD19 chimeric antigen receptor T-cell therapy for children, adolescents, and young adults. Open Forum Infect Dis. 2020;7(5):ofaa121. 32432149 10.1093/ofid/ofaa121 PMC7221263

[6] Busca A Salmanton-García J Corradini P. COVID-19 and CAR T cells: a report on current challenges and future directions from the EPICOVIDEHA survey by EHA-IDWP. Blood Adv. 2022;6(7):2427–2433. 34749396 10.1182/bloodadvances.2021005616 PMC8575532

[7] Committee of Neoplastic Supportive-Care, China Anti-Cancer Association; Cancer Clinical Chemotherapy Committee of China Anti-Cancer Association. Chinse expert consensus on issues related to the protection, treatment and management of patients with solid tumors during COVID-19 (2022 edition). Zhonghua Zhong Liu Za Zhi. 2022;44(10):1083–1090. Chinese. 36319453 10.3760/cma.j.cn112152-20220505-00309

[8] NCCN. Prevention and Treatment of Cancer-Related infections, Version 3.2022, NCCN. Clinical Practice Guidelines in Oncology. Available from: https://www.nccn.org/guidelines/guidelines-detail?category=3&id=1457. 10.6004/jnccn.2024.0056 39536464

[9] Zhao H Ye W Yu X Shi Y Sheng J. Omicron COVID-19 variant outcomes and vaccination in non-severe and non-critical patients at admission. Front Public Health. 2023;10:974986. 36845347 10.3389/fpubh.2022.974986 PMC9948012

[10] Ullrich F Hanoun C Turki AT. Early report on the severity of COVID-19 in hematologic patients infected with the SARS-CoV2 omicron variant. Eur J Haematol. 2022;109(4):364–372. 35751531 10.1111/ejh.13818 PMC9350268

[11] Rahmani S Rezaei N. SARS-CoV-2 Omicron variant: why global communities should take it seriously? Immun Inflamm Dis. 2022;10(5):e618. 35478443 10.1002/iid3.618 PMC9017627

[12] Shah GL DeWolf S Lee YJ. Favorable outcomes of COVID-19 in recipients of hematopoietic cell transplantation. J Clin Invest. 2020;130(12):6656–6667. 32897885 10.1172/JCI141777 PMC7685738

[13] McNerney KO Richards RM Aguayo-Hiraldo P. SARS-CoV-2 infections in pediatric and young adult recipients of chimeric antigen receptor T-cell therapy: an international registry report. J ImmunoTher Cancer. 2023;11(1):e005957. 36707090 10.1136/jitc-2022-005957 PMC9884906

[14] Campana D van Dongen JJ Mehta A. Stages of T-cell receptor protein expression in T-cell acute lymphoblastic leukemia. Blood. 1991;77(7):1546–1554. 1826223

[15] Gomes-Silva D Srinivasan M Sharma S. CD7-edited T cells expressing a CD7-specific CAR for the therapy of T-cell malignancies. Blood. 2017;130(3):285–296. 28539325 10.1182/blood-2017-01-761320 PMC5520470

[16] Fredricks DN. The gut microbiota and graft-versus-host disease. J Clin Invest. 2019;129(5):1808–1817. 31042160 10.1172/JCI125797 PMC6486325

[17] Jiang X Luo M Zou Z Wang X Chen C Qiu J. Asymptomatic SARS-CoV-2 infected case with viral detection positive in stool but negative in nasopharyngeal samples lasts for 42 days. J Med Virol. 2020;92(10):1807–1809. 32330309 10.1002/jmv.25941 PMC7264508

[18] Wang JG Cui HR Tang HB Deng XL. Gastrointestinal symptoms and fecal nucleic acid testing of children with 2019 coronavirus disease: a systematic review and meta-analysis. Sci Rep. 2020;10(1):17846. 33082472 10.1038/s41598-020-74913-0 PMC7576139

[19] Ziegler CGK Allon SJ Nyquist SK. SARS-CoV-2 receptor ACE2 is an interferon-stimulated gene in human airway epithelial cells and is detected in specific cell subsets across tissues. Cell. 2020;181(5):1016–1035.e19. 32413319 10.1016/j.cell.2020.04.035 PMC7252096

[20] Baindara P Chakraborty R Holliday ZM Mandal SM Schrum AG. Oral probiotics in coronavirus disease 2019: connecting the gut-lung axis to viral pathogenesis, inflammation, secondary infection and clinical trials. New Microbes New Infect. 2021;40:100837. 33425362 10.1016/j.nmni.2021.100837 PMC7785423

[21] Ali H Ngo D Aribi A. Safety and tolerability of SARS-CoV2 emergency-use authorized vaccines for allogeneic hematopoietic stem cell transplant recipients. Transplant Cell Ther. 2021;27(11):938.e1–938.e6. 10.1016/j.jtct.2021.07.008 PMC8280601 34274492

[22] Ngo D Chen J Tinajero J. The impact of SARS-CoV2 vaccines on the incidence of graft versus host disease in allogeneic hematopoietic stem cell transplant recipients: a single-center retrospective study. Stem Cell Res Ther. 2023;14(1):95. 37072867 10.1186/s13287-023-03326-3 PMC10112306

[23] Yu B Chang J. The first Chinese oral anti-COVID-19 drug Azvudine launched. Innovation (Camb). 2022;3(6):100321. 36106026 10.1016/j.xinn.2022.100321 PMC9461232

