# Supplementary material for: Acute intestinal GVHD following donor-derived CD7-CAR-T-cell infusion in a child with Omicron COVID-19
Source: Blood Sci. 2023 Nov 2;5(4):269–73. doi: 10.1097/BS9.0000000000000170 (PMC10629738; doi:10.1097/BS9.0000000000000170)

Acute intestinal GVHD following donor-derived CD7-CAR-T-cell infusion in a child with Omicron COVID-19

Supplemental Material

Supplemental Table 1 2

Supplemental Figure 1 3

Supplemental Table 1 Key events of the patients.

Time before and after CAR T cell therapy	Date	Key events
`-2.5 years	2020/6	Diagnosis of T-ALL
`-2.5 to -1 years	2020/7 to 2021/9	8 cycles of chemotherapy and sustained remission
- 1 year	2021/11/16	haploidentical hematopoietic stem cell transplantation(father, 6/12 HLA match)
`-3 months to -1 months	2022/8 to 2022/10	extramedullary and bone marrow relapse
-4 days to -2 days	2022/11/18 to 2022/11/20	Standard lymphodepleting chemotherapy regimen
**0 days**	**2022/11/22**	**Infusion of donor-derived CD7-CAR-T-cell therapy**
23 days	2022/12/15	infection with Omicron variant
27 days	2022/12/19	acute graft versus host disease((gut, grade III; liver, grade III)
85 days	2023/2/15	positive for Omicron variant again

Supplement Figure 1 Morphological evaluation of bone marrow（Giemsa 1000×）4 days before(a) and 3 months after(b) donor-derived CD7-CAR-T-cell therapy.

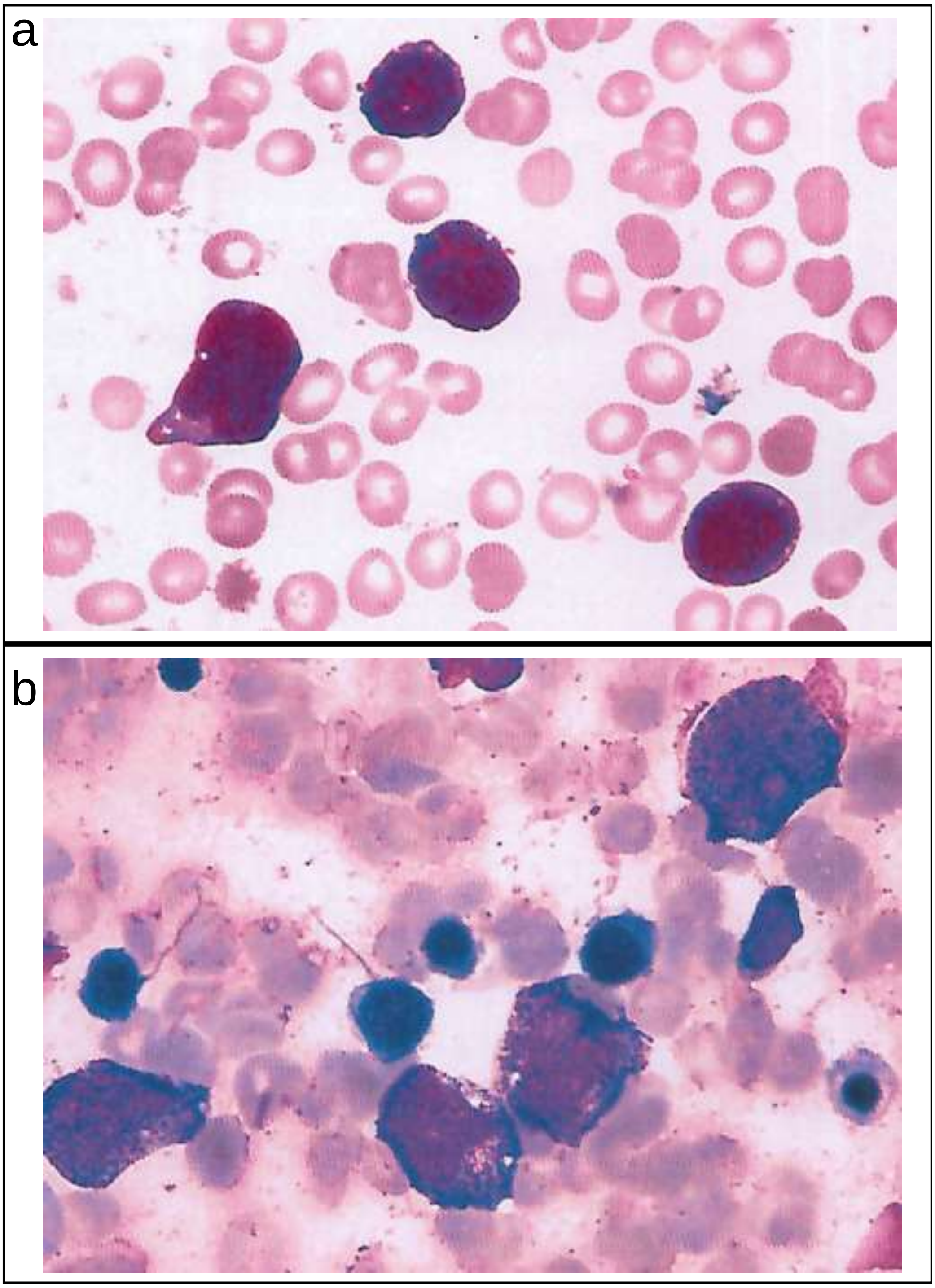

Supplement: Supplementary file 1 [file bs9-5-269-s001.pdf]
